# Pretransplant Comorbidities Maintain Their Impact on Allogeneic Stem Cell Transplantation Outcome 5 Years Posttransplant: A Retrospective Study in a Single German Institution 

**DOI:** 10.1155/2014/853435

**Published:** 2014-03-05

**Authors:** Jens M. Chemnitz, Geothy Chakupurakal, Maya Bäßler, Udo Holtick, Sebastian Theurich, Alexander Shimabukuro-Vornhagen, Silke Leitzke, Michael S. Von Bergwelt-Baildon, Christof Scheid

**Affiliations:** BMT Program, Department I of Internal Medicine, University Hospital Cologne, Kerpener Straße 62, 50924 Köln, Germany

## Abstract

The introduction of reduced-intensity conditioning regimens has allowed elderly patients with preexisting comorbidities access to the potentially curative allogeneic stem cell transplantation. Patient's comorbidities at the time of treatment consideration play a significant role in transplant outcome in terms of both overall survival (OS) and nonrelapse mortality (NRM). The hematopoietic stem cell transplantation comorbidity index (HCT-CI) quantifies these patient specific risks and has established itself as a major tool in the pretransplant assessment of patients. Many single center and multicenter studies have assessed the HCT-CI score and reported conflicting outcomes. The present study aimed to evaluate the HCT-CI in a single large European transplant centre. 245 patients were retrospectively analyzed and the predictive value of the score was assessed with respect to OS and NRM. We confirm that the HCT-CI predicts outcome for both OS and NRM. Moreover, we identified age of the patient as an independent prognostic parameter for OS. Incorporation of age in the HCT-CI would improve its ability to prognosticate and allow the transplant physician to assess the patient specific risks appropriately at the time of counseling for transplant.

## 1. Introduction

Allogeneic hematopoietic stem cell transplantation (HSCT) is a curative therapeutic option for a variety of haematological malignancies [1]. As the average life expectancy increases each year, more and more elderly patients are diagnosed with cancer and therapeutic modalities need to be modified to cater the needs of this patient cohort. Conventional myeloablative conditioning regimens cannot be offered to patients above 55 with comorbidities due to its regimen related toxicities, resulting in turn in high nonrelapse mortality. Reduced intensity conditioning regimens take advantage of the graft-versus-leukemia (GvL) effect of the donor cells without eradicating the leukaemia clone with high dose therapy and offer a safer therapeutic option for this elderly cohort of patients [2, 3]. To balance treatment related risks with the influence of preexisting patient specific comorbidities [4] different assessment tools have been developed to guide patient counseling before allogeneic stem cell transplantation especially for elderly patients.

The Charlson Comorbidity Index (CCI) has been used to predict treatment-related mortality (TRM) risks for various solid tumours by assigning weights for 19 chronic conditions based on their association with mortality [5, 6]. Lack of inclusion of significant comorbidities like preexisting infections and stringent pretransplant exclusion criteria meant that in the HSCT setting CCI had very low sensitivity to identify the patients at a higher risk of a TRM. This leads to the development of hematopoietic cell transplantation-comorbidity index (HCT-CI) [7]. This score was developed based on 1055 patients treated with various nonmyeloablative (*n* = 294) or ablative (*n* = 761) conditioning regimens in a single institution, Fred Hutchinson Cancer Research Center (FHCRC). HCT-CI score covers 17 different comorbidities with different integer weights between 1 and 3 assigned to each. The authors found that the HCT-CI score was more representative of the patient cohort considered for a transplant and provided a better assessment of nonrelapse mortality (NRM) and overall survival (OS) risks compared with the original CCI. Retrospective reviews performed in various institutions gave conflicting reports [8–14]. This retrospective study aims to assess the ability of HCT-CI to predict outcome with respect to OS and NRM in a large German single center transplant unit, University of Cologne, Germany.

## 2. Patients and Methods

### 2.1. Patients

We retrospectively analyzed all patients treated with HSCT between 2000 and 2009 at our Stem Cell Transplant Unit, University Hospital of Cologne, Germany. All consecutive patients identified within the timeframe, irrespective of the underlying disease and conditioning regimen, were included in the study. All patients gave their informed consent to the planned treatment schedule as well as to anonymized data collection and analysis. Antibiotics were routinely administered as prophylaxis against bacterial (Ciprofloxacin), fungal (Fluconazole), pneumocystis carinii (Pentamidine), and herpes virus (Aciclovir) infections. Early detection of cytomegalovirus antigenemia by twice weekly screening and preemptive ganciclovir therapy, in patients with early signs of reactivation, were routinely performed in all patients.

### 2.2. Comorbidity Assessment

All relevant investigations were performed within the routine workup for transplant. A questionnaire was developed based on the HCT-CI scoring system [7] and data was extracted from the medical records as well as laboratory values at the time of transplant. Comorbidities of each patient were scored according to the HCT-CI on the worksheet. The final score obtained for each patient was then correlated with available data on our database.

### 2.3. Statistics

Results were analyzed as of November 30, 2013. Overall survival (OS) was defined as time to death from transplantation irrespective of cause. Nonrelapse mortality (NRM) was defined as time to death from transplant without evidence of disease relapse or recurrence. Survival curves for OS and NRM were estimated by the Kaplan-Meier method and differences tested by log rank test. A two-sided *P* value of <0.05 was considered significant. Multivariate analysis was performed using a Cox proportional hazard model. All statistical analyses were performed using the SPSS-21 software.

## 3. Results

### 3.1. Patient Characteristics

We identified 245 patients, 109 female and 136 male, who consecutively received an allogeneic HSCT between 2000 and 2009 in our institution. The demographic data is summarised in Table 1. Median age of the patients was 45 years, (range 18–68 years). Only 6 patients (2%) were younger than 20 years and 27 patients (11%) older than 61 years. The most frequent haematological disease was AML with 99 patients (40%), followed by ALL (*n* = 45; 18%). 14.6% were lymphoma patients, whereas MDS/secondary AML, CML, and CLL each represented less than 20% of the total. Multiple myeloma, myeloproliferative neoplasms, and severe aplastic anaemia accounted for less than 5% each. 80 patients (32.7%) received a myeloablative conditioning regimen, whereas the remaining received nonmyeloablative regimens of varying intensities ranging from FLAMSA/TBI to Flu TBI. The majority (158/245, 64.5%) received an unrelated donor transplant and peripheral blood stem cells (230/245, 94%). After a mean follow-up period of 65 months (maximum of 159 months), 169/245 patients died with an OS of 31% of whom 69/245 (28%) died of disease and in 88/245 (36%) death was not disease related.

### 3.2. HCT-CI

**Figure 1:**
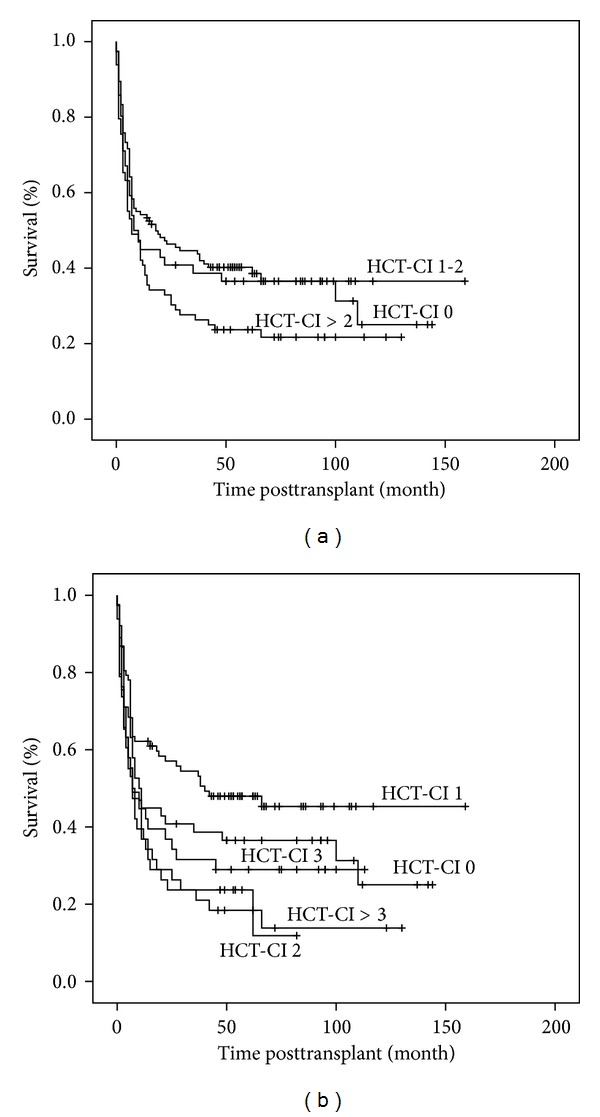
Overall survival based on HCT-CI score. (a) Patients are categorised into three risk groups: HCT-CI 0 = low risk; HCT-CI 1-2 = intermediate risk; HCT-CI > 2 = high risk. (b) Patients are categorised into five risk groups HCT-CI 0, 1, 2, 3, and >3.

**Figure 2:**
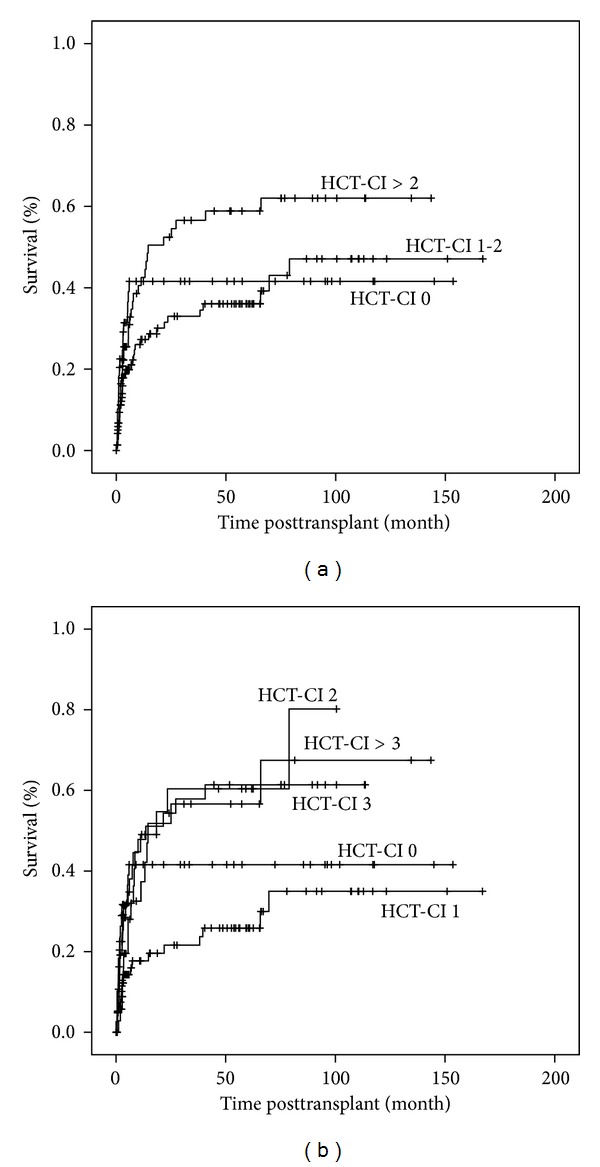
Nonrelapse mortality based on HCT-CI. (a) Patients are categorised into three risk groups: HCT-CI 0 = low risk; HCT-CI 1-2 = intermediate risk; HCT-CI >2 = high risk. (b) Patients are categorised into five risk groups HCT-CI 0, 1, 2, 3, and >3.

The patients could be classified into eight groups based on their HCT-CI score (Table 2). 49 patients (20.0%) had no comorbidities, 33.5% had a score of 1, 15.5% had a score of 2. 76 (31%) patients were assigned a score of 3 or more. The patients were subdivided into three cohorts as originally proposed by Sorror: low risk (HCT-CI 0, *n* = 49), intermediate risk (HCT-CI 1-2, *n* = 120), and high risk (HCT-CI > 2, *n* = 76). The high risk group showed a trend for an inferior OS while the curves for low and intermediate risk did not separate and the difference between the groups was not statistically significant (*P* = 0.12) (Figure 1(a)). This led us to classify the patients into 5 groups based on the HCT-CI score; 0, 1, 2, 3, and above 3, within which the different groups had a statistically significant OS (*P* = 0.008) (Figure 1(b)). Similar results were obtained for NRM. The original Sorror risk group categories (Figure 2(a)) were not found to be significant (*P* = 0.096), whereas the five subgroups which we categorised had a statistically significant difference with respect to NRM over the same observation period (*P* = 0.009) (Figure 2(b)). A different subgrouping into 1 = HCT-CI 0 and 1, 2 = HCT-CI 2 and 3, and 3 = HCT CI > 3 was also found to be statistically significant (*P* = 0.006) which was not observed in the OS analyses.

**Figure 3:**
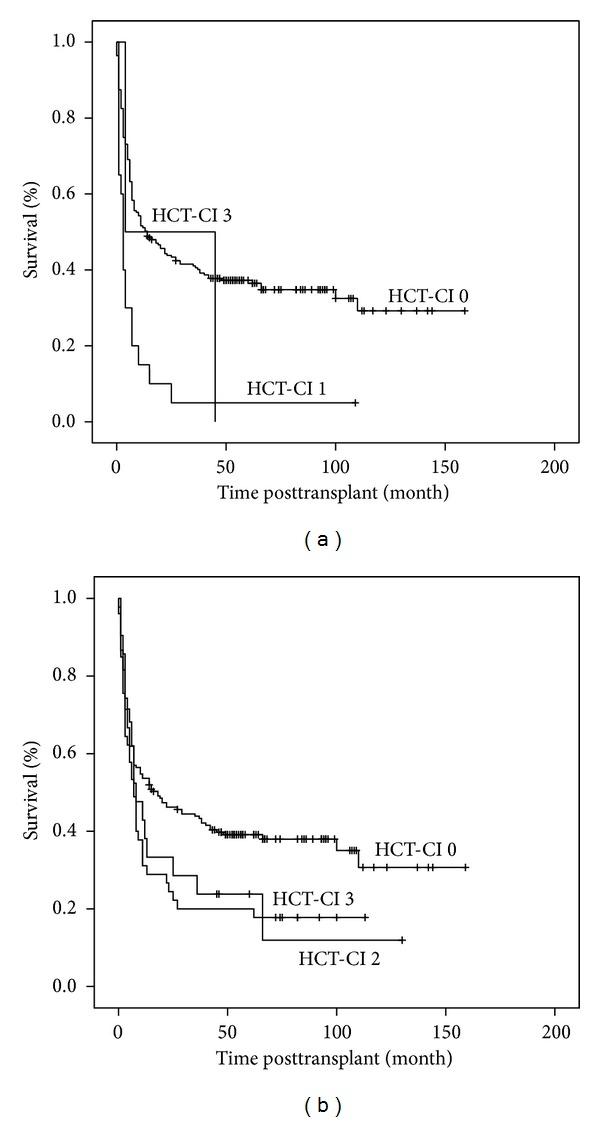
Overall survival based on scores assigned for (a) cardiac or (b) pulmonary comorbidities.

Cardiac, pulmonary, and hepatic comorbidities were most commonly observed within the study population. In the HCT-CI scoring system, patients with a history of cardiac arrhythmias, coronary vascular disease, myocardial infarction or congestive heart failure, or an ejection fraction below 50% are given a score of 1, whereas heart valve disease of grade 3 or 4 excluding mitral valve prolapse is given a score of 3. In our cohort 20/245 (8%) patients scored 1 due to cardiac comorbidities and 2/245 (0.8%) with heart valve disease had a score of 3. 19 of the 20 patients with a score of 1 and both patients with a score of 3 died during followup highlighting the significant role that cardiac comorbidities play in relation to transplant outcome (*P* < 0.001) (Figure 3(a)).

HCT-CI score allows the classification of the patients based on their lung function tests into three groups irrespective of the underlying cause. Moderate pulmonary comorbidity defined as diffuse lung capacity (DLCO) and/or FEV1 66–80% or dyspnoea on slight activity allocates a score of 2 and severe pulmonary comorbidity defined as (DLCO) and /or FEV1 ≤ 65% or dyspnoea at rest or oxygen requirement allocates a score of 3. Mild pulmonary comorbidity is defined as (DLCO) and/or FEV1 81–90% or dyspnoea at moderate activity is not included in the HCT-CI scoring system. We analysed the impact of pulmonary function prior to transplant on transplant outcome. 179/245 (73%) patients had a normal lung function prior to transplantation (score 0), 45 a moderate pulmonary comorbidity (score 2), and 21 severe pulmonary comorbidity (score 3). Pulmonary comorbidity had a statistically significant influence on overall survival (*P* = 0.020) (Figure 3(b)) with no apparent difference between HCT-CI scores 2 or 3.

17 (7%) patients had a mild hepatic comorbidity (score 1) (bilirubin > ULN to 1.5 × ULN or AST/ALT > ULN to 2.5 × ULN) and 1 patient additional liver cirrhosis with portal hypertension (score 3). 6/17 patients with a score of 1 and the patient with score 3 died during followup. In our cohort hepatic comorbidity was not associated with a statistically significant impact on OS after transplant. 27 (11%) patients had a prior malignancy; however this did not influence OS following transplant. A preexisting infection requiring treatment at day 0 was identified in 124 (50%) patients giving these patients a score of 1. After transplant more patients (92/121, 76%) died in the subgroup without infections, that is, with a score of 0 in comparison to the group with infections (72/124, 58%). Lack of influence of preexisting infections requiring treatment on OS may be because of the longer duration of followup and the influence of the underlying disease as well as other comorbidities on outcome.

Comorbidities related to inflammatory bowel disease, diabetes, cerebrovascular disease, psychiatric disturbance, peptic ulcer, obesity, preexisting rheumatologic disease, or renal impairment were infrequent and not associated with a significant impact on OS.

### 3.3. Influence of Age

**Figure 4:**
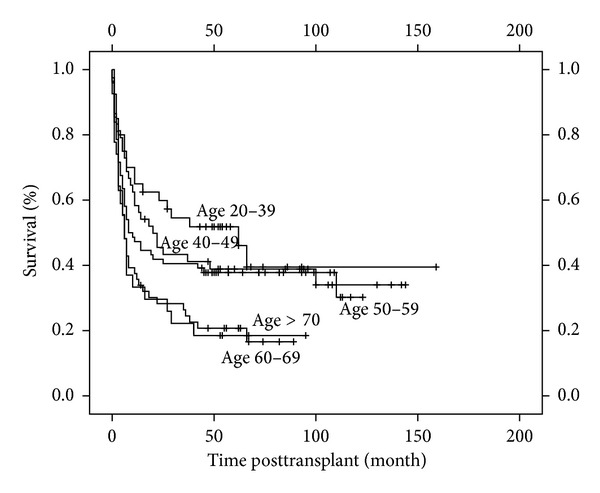
Overall survival based on HCT-CI scores and age. Patients are classified into different age groups from 20–39, 40–49, 50–59, 60–69, and >70.

Age alone is an important factor that influences the decision to transplant as well as the conditioning regimen employed [15, 16]. We wanted to evaluate the influence of age on OS in relation to the HCT-CI score. The study cohort was subdivided into different age groups. As shown in Table 3, most patients were distributed between 21 and 40 years (*n* = 88, 36%) and between 41 and 50 (*n* = 74, 30%). 51/88 (58%) patients, within the age group of 20–40 years, died during followup. 22/27 (81%) patients older than 61 died during followup. We observed a distinct reduction in the median OS for each age group with patients under 31 years of age achieving the maximum of 62 months and those in the age group above 61 years surviving up to a median of 6 months (Figure 4).

Next, we allocated the patients within the different age groups to the calculated HCT-CI scores, HCT-CI low/intermediate with a score of 0–2, and high risk with a score above 2. HCT-CI of 0–2 was more frequent in each age group (Table 3). The mortality for patients with a HCT-CI >2 was above 50% within each age group. The median OS of each age group scoring HCT-CI >2 decreased from 11 months for patients between 20 and 31 to 6 months for patients between 61 and 70.

## 4. Discussion

Increase in the average age of the cancer patients has led to a concerted effort in developing scoring systems which help in predicting outcomes following treatment [17]. HCT-CI has established itself in this setting as a reliable tool to predict outcome following HSCT. Our retrospective study confirms the role of HCT-CI to predict OS and NRM of the transplant patients and shows that this impact persists over a longer period of time. The original risk group classification suggested by the Sorror group showed a distinct trend (however not statistically significant), whereas a classification into 5 groups showed a significant impact on OS as well as NRM. Cardiac and pulmonary comorbidities were frequent and associated with a significantly inferior OS similar to that described by other groups [12]. In addition to the impact of HCT-CI, we have also shown that age is an independent predictor of OS in this cohort of patients.

Sorror et al. [7] developed the HCT-CI score using a patient cohort of 1055 patients 708 included in the training set and 347 in the validation set. The median age and sex distribution of our group are comparable to their validation set. 20.7% in our cohort had a HCT-CI score of 0 compared to 38% in their cohort. The intermediate risk group comprised 49% of the patients in our cohort compared to 34% in their group, whereas the high risk group was 31% of our cohort and 28% of the total in the cohort by Sorror et al. (Table 4). Though the prevalence of cardiac and pulmonary comorbidities, 9% and 27%, respectively, in our group was comparable to the Sorror group, 7% and 34%, respectively, 20% of the patients in the Sorror group had a hepatic comorbidity compared to 7% in our group. The prevalence of preexisting infections was significantly higher in our patient population, 58% compared to 4% in the Sorror group. This can be attributed to a liberal definition of infections treated on day 0 as well as the inclusion of patients who continued antifungal treatment despite a good response, due to a persistent risk of recurrence. Rare comorbidities like inflammatory bowel disease, cerebrovascular disease, obesity, peptic ulcer, and rheumatologic comorbidities were similarly distributed in both cohorts.

Birninger et al. [18] conducted a single center retrospective study in another German transplant centre, Dresden, focusing exclusively on patients with high risk acute myeloid leukemia. Though their group had a median age and sex distribution comparable to our cohort, 74% of the patients were assigned a high risk HCT-CI score (≥3) compared to 31% in our group. They included patients with grade 1 and grade 2 heart valve insufficiency resulting in 44% of the patients being assigned an HCT-CI score 3 based on heart valve disease alone. With a median followup of 30 months, they found no predictive value of the HCT-CI for either OS or NRM. Though the practices within the German transplant network are similar between the patient groups our cohort includes a very heterogeneous group of patients including different diseases. Only 9% of our patient cohort had an HCT-CI score resulting from cardiac involvement. These differences may explain why we have found a significant correlation between HCT-CI score pretransplant and OS and NRM following transplant.

Age was excluded from the HCT-CI score as a comorbidity, as it is already an exclusion criteria for the transplant and crucial in deciding the conditioning regimen but was used to adjust the Cox regression hazards. The influence of age on transplant outcome has been disputed [19–22]. More than age alone the combination of age along with severe comorbidities and severe functional impairment influence transplant outcome [23]. Our analysis identified age as a significant factor which independently influences outcome. It may be pertinent to include an integer for age and thereby modify the HCT-CI score increasing its predictive capacity.

A big drawback of our data is the possibility of erroneous scoring which could have incurred due to false subjective interpretation during retrospective data collection. It is possible that comorbidities such as inflammatory bowel disease, cerebrovascular disease, obesity, peptic ulcer, and rheumatologic comorbidities were not documented and hence missed out resulting in a wrong final score. Wrong scoring based on subjective diagnosis criteria, for example, by assessing infectious or psychiatric comorbidities cannot be excluded. Thus, a strict and comprehensible web based calculation tool used prospectively is clearly helpful for standardized evaluation of the patients as suggested by Sorror et al. [24]. GvHD as an independent predictor has not been analysed due to unavailability of data which again is a drawback of our data analysis.

In conclusion we performed a retrospective analysis on a large single center patient cohort aiming to assess the HCT-CI as a predictive tool for OS and NRM post HSCT. HCT-CI was found to predict outcome for both OS and NRM, thereby representing a helpful instrument in patient counselling. The HCT-CI was developed on a patient cohort observed over a 2-year period. We observe the impact of pretransplant comorbidities persisting even after 65 months, further highlighting the importance of comorbidities on outcome. Possible errors in scoring the patients cannot be excluded and can be minimized by using a web based tool as well as prospective data collection.

## Figures and Tables

**Table 1:** Patient characteristics.

Characteristic	*N* (%)
Male/female	136/109 (56/44)
Age	
<31	40 (16.3)
31–40	48 (19.6)
41–50	74 (30.2)
51–60	56 (22.8)
61–70	27 (11)
Diagnoses	
AML	98 (40.2)
ALL	45 (18.3)
MDS/sAML	13 (5.3)
CML	19 (7.7)
CLL	15 (6.1)
NHL	18 (7.3)
HD	18 (7.3)
MM	8 (3.3)
SAA/FA	7 (2.8)
MPN	4 (1.6)
Conditioning regimen	
BuCy	44 (18)
Cyc/TBI	27 (11)
TBI/VP16	9 (3.7)
FLAMSA/TBI	42 (17.1)
FLU/MEL140	23 (9.4)
FLU/TREO	16 (6.5)
FLAMSA no TBI	6 (2.4)
TBI 2 Gy	7 (2.9)
TBI 2 Gy/FLU	28 (11.4)
Other	43 (17.6)
Donor type	
Related	87 (35.5)
Unrelated	158 (64.5)
Cell source	
PBMC	230 (94)
Marrow	12 (4.9)
N.a.	3 (1.1)
Prior HSCT	
No prior HSCT	223 (91)
Prior autologous PBSC	22 (9)

AML: acute myeloid leukaemia; ALL: acute lymphocytic leukaemia; MDS/sAML: myelodysplastic syndrome/secondary AML; CML: chronic myeloid leukaemia; CLL: chronic lymphocytic leukaemia; NHL: non-Hodgkin's lymphoma; HD: Hodgkin's lymphoma; MM: multiple myeloma; SAA/FA: severe aplastic anemia/fanconi anemia; MPN: myeloproliferative neoplasm; Bu: busulfan, Cy: cyclophosphamide, TBI: total body irradiation, VP16: etoposide, FLAMSA: fludarabine, Ara-C and Amsacrine, Mel: melphalan, Flu: fludarabine, Treo: treosulfan, PBMC: peripheral blood mononuclear cells; HSCT: hematopoietic stem cell transplantation.

**Table 2:** Risk groups based on HCT-CI score.

	Number (%)
HCT-CI score	
0	49 (20)
1	82 (33.5)
2	38 (15.5)
3	38 (15.5)
4	20 (8.2)
5	12 (4.9)
6	5 (2)
7	1 (0.4)
Risk groups according to HCT-CI	
Low (score 0)	49 (20)
Intermediate (score 1-2)	120 (49)
High (score >2)	76 (31)

**Table 3:** Risk groups based on HCT-CI and age along with the OS of each subgroup.

Age groups	HCT-CI	*N* (%)	Median OS (months)	Events
20–30	0–2	34 (85.0)	62	17
	>2	6 (15.0)	11	4
	Total	40	62	21

31–40	0–2	37 (77.1)	22	23
	>2	11 (22.9)	13	7
	Total	48	20	30

41–50	0–2	44 (59.5)	8	26
	>2	30 (40.5)	8	21
	Total	74	8	47

51–60	0–2	36 (64.3)	6	26
	>2	20 (35.7)	5	19
	Total	56	6	45

61–70	0–2	18 (66.7)	6	14
	>2	9 (33.3)	6	8
	Total	27	6	22

**Table 4:** Comparison of data with Sorror et al. [7] and Birninger et al. [18].

	Chemnitz et al.	Sorror et al. [7]	Birninger et al. [18]
	245	347	370 (only 340 included)
Median age	45	44.5	53
Related donors (%)	35.5	58	34.1
HCT-CI (low 0)	49 (20%)	132 (38%)	18 (5%)
HCT-CI (intermediate 1, 2)	120 (49%)	118 (34%)	70 (21%)
HCT-CI (High ≥3)	76 (31%)	97 (28%)	252 (74%)
Prevalence of pulmonary comorbidity	27%	34%	34%
Prevalence of cardiac comorbidity	9%	7%	56%
Prevalence of hepatic comorbidity	7%	20%	51%
